# Estimates of delays in diagnosis of cervical cancer in Nepal

**DOI:** 10.1186/1472-6874-14-29

**Published:** 2014-02-17

**Authors:** Deepak Gyenwali, Gita Khanal, Rajan Paudel, Archana Amatya, Jitendra Pariyar, Sharad Raj Onta

**Affiliations:** 1 Department of Community Medicine and Public Health, Institute of Medicine, Tribhuvan University, Kathmandu, Nepal; 2 Department of Nursing, Bharatpur Hospital, Chitwan, Nepal; 3 Gynecologic Oncology Unit, B.P. Koirala Memorial Cancer Hospital, Chitwan, Nepal

**Keywords:** Alarming symptoms, Cervical cancer, Delays, Health care provider, Nepal

## Abstract

**Background:**

Cervical cancer is the leading cause of cancer related deaths among women in Nepal. The long symptom to diagnosis interval means that women have advanced disease at presentation. The aim of this study was to identify, estimate and describe the extent of different delays in diagnosis of cervical cancer in Nepal.

**Methods:**

A cross-sectional descriptive study was conducted in two tertiary cancer hospitals of Nepal. Face to face interview and medical records review were carried out among 110 cervical cancer patients. Total diagnostic delay was categorized into component delays: patient delay, health care providers delay, referral delay and diagnostic waiting time.

**Results:**

Total 110 patients recruited in the study represented 40 districts from all three ecological regions of the country. Median total diagnostic delay was 157 days with more than three fourth (77.3%) of the patients having longer total diagnostic delay of >90 days. Out of the total diagnostic delay, median patient delay, median health care provider delay, median referral delay and median diagnostic waiting time were 68.5 days, 40 days, 5 days and 9 days respectively. Majority of the patients had experienced longer delay of each type except referral delay. Fifty seven percent of the patients had experienced longer patient delay of >60 days, 90% had suffered longer health care provider delay of >1 week, 31.8% had longer referral delay of >1 week and 66.2% had waited >1 week at diagnostic center for final diagnosis. Variation in each type of delay was observed among women with different attributes and in context of health care service delivery.

**Conclusions:**

Longer delays were observed in all the diagnostic pathways except for referral delay and diagnostic waiting time. Among the delays, patient delay is of crucial importance because of its longer span, although health care provider delay is equally important. In the context of limited screening services in Nepal, the efforts should be to reduce the diagnostic delay especially patient and health care provider delay for early detection and reduction of mortality rate of cervical cancer.

## Background

Cervical cancer is the third most commonly diagnosed cancer in women worldwide with more than 85.0% burden in developing countries [1]. Although, cervical cancer can be cured if detected at earlier stage [2,3], it continues to be a major public health threat to women in Nepal where it is still the leading cancer with high morbidity and mortality [1,4]. With an incidence rate of 32 per 100,000 per annum and mortality about 18 per 100,000 per annum it accounts 21.0% of total female cancer in Nepal [5,6].

One of the most important prognostic factors for cervical cancer is how early the disease is detected and how far it has spread [7,8]. Early diagnosis of cancer results in lower stages of the cancer, less intensive treatment and improved survival [7,9,10]. In Nepal, most of the cervical cancer patients have been reported diagnosed at advanced stage [11,12] indicating the long duration between disease onset and final diagnosis of the disease.

Diagnostic delay covers the period from the patient’s first experience of symptoms until diagnosis [13]. Reducing diagnostic delay may increase the proportion of early stage cancers and improve survival [7]. Delays may occur at different stages of the cancer diagnostic journey and have been commonly defined as being either patient focused or healthcare provider focused [7,13]. Commonly, delay is found further categorized into different component delays such as patient delay, health care provider delay, referral delay and system delay [7,13,14]. Delays are calculated on the basis of dates provided by the patients and/or health care providers. Each type of delays have been found influenced by different conditions and characteristics related to either patients or health care providers or service delivery system [13,15-19].

Knowledge of delays is crucial in cancer prevention and control and it has been the subject of research for decades in developed countries. In Nepal, this issue has not been given much of importance and there is lack of researches related to this issue. The aim of this study was to identify, estimate and describe the extent of different delays in cervical cancer diagnosis in Nepalese context.

### Operational definitions

#### ***Symptoms***

Symptoms were defined as any patient’s complaints that led to a diagnosis of cervical cancer and included foul smelling vaginal discharge, lower abdominal pain, back ache and abnormal bleeding per vaginum.

### Patient delay

The time period from a patient first becoming aware of symptoms till their first presentation to a health care provider (HCP). The duration of more than 60 days was defined as “long patient delay” and 60 days or less was defined as “short patient delay” [13].

### Health care provider’s (HCP) delay

The time period between patient’s first presentation to the health care provider (HCP) and the final referral by HCP to the cancer diagnostic center. The period of seven days or less was defined as “short HCP delay” and more than seven days was referred as “long HCP delay”.

### Referral delay

The time interval between the date of final referral by health care provider to diagnostic center with suspicion of cervical cancer and the date of first appointment of patient in the cervical cancer diagnostic center. The period of seven days or less was defined as “short referral delay” and more than seven days was referred as “long referral delay”. Actually, it is patient’s decision making period and the travel period to reach the diagnostic center after referral by HCP.

### Diagnostic waiting time

This includes waiting time for all relevant investigations of symptoms in the diagnostic center. The period of seven days or less was defined as “short waiting time” and more than seven days was defined “long waiting time”.

### Total diagnostic delay

The time period between onset of symptoms of cervical cancer and confirmed diagnosis. The period of more than 90 days was defined as “long diagnostic delay” and 90 days or less as “short diagnostic delay” [20].

[Total diagnostic delay = patient delay + health care provider delay + referral delay + diagnostic waiting time.]

In this study, the term “delay” refers to the time interval between two specific events in diagnostic pathway. Because there is no standard cutoff point to dichotomize the interval into “short delay” and “long delay; and it is contextual, such cutoff points for different delays were defined in context of socio-cultural aspect, health seeking behavior of women and health care system of Nepal.

## Methods

**Figure 1:**
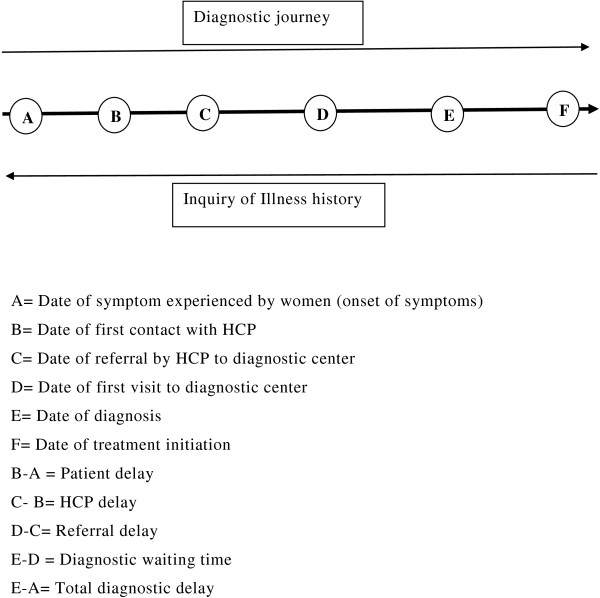
Concept of delays and way of history taking.

A cross- sectional descriptive study was carried out at two cancer hospitals of Nepal: B.P. Koirala memorial cancer hospital (BPKMCH), Bharatpur, Chitwan and Bhaktapur cancer hospital, Bhaktapur from August to October, 2012. These are the only two referral cancer hospitals which cater the most of the cancer diagnosis and treatment services in Nepal [11]. Nepali women diagnosed of cervical cancer for the first time and attending the hospitals during study period were included in the study. Critically ill and patients diagnosed outside Nepal were excluded. Numbers of participants were selected proportionately on the basis of case load in hospitals. According to hospital records, 505 cases in BPKMCH and 122 cases in Bhaktapur cancer hospital were diagnosed of cervical cancer in 2010. Out of 110 sample cases, 90 cases were taken from BPKMCH and 20 cases from Bhaktapur cancer hospital. To reduce selection bias, data collection was done on alternate days during study period. Cervical cancer patients attending to the hospitals were identified from the registration department in each day of data collection. Then all the available patients meeting the inclusion criteria were invited to participate in the study. Face to face interview with patients was conducted using pre-tested structured questionnaire for the socio-demographic information and information on history of diagnostic journey. Patient’s medical documents were reviewed for supplementary information regarding different dates in diagnosis. The concept of delay in diagnostic journey and ways of inquiring illness history of patients is elaborated in Figure 1. Dates used for estimating delays were date of symptoms experienced, date of first consultation with health care provider (HCP), date of final referral to cancer diagnostic center by HCP, date of first visit to cancer diagnostic center and date of confirmed diagnosis. The illness history was taken in a retrospective manner starting from the most recent event in diagnostic pathway i.e. date of diagnosis or treatment initiation of cervical cancer and probing backward without interruption until the information about the type and date of symptoms experienced (Figure 1). If the patients were unable to recall the exact date of symptoms experienced and date of first consultation with HCP, such dates were approximated by probing the exact week of the respective month and year of symptoms experienced and first consultation with HCP. The mid-point of that week was approximated as date of symptoms experienced and date of first consultation with HCP accordingly. Analysis of data was done by using computer software SPSS 18.0 version. Delays in diagnosis were measured in terms of continuous variables i.e. days.

Interviewers explained the participants the purpose of the study, obtained informed consent from the eligible participants and interviewed them individually in a separate room. The study protocol and questionnaire were approved for ethical clearance by the board of thesis committee of Institute of Medicine, Tribhuvan University. In addition, permission was obtained from both hospitals before starting the study.

## Results

### Characteristics of the patients of cervical cancer

Out of 75 districts of Nepal, the study participants had represented 40 districts of all three ecological regions: plain, hill and mountain. Among the participants, 75 women were diagnosed in cancer hospitals and 35 women in other hospitals of Nepal. Selected characteristics of the research participants are presented in Table 1. The mean age of the participants was 52.72 years (SD = 10.63), the youngest patient was 27 years and the oldest 79 years. Two third (66.4%) of the patients were illiterate and most of them (77.3%) were from rural areas. Eighty percent of the participants were currently married and living together while 20.0% were widows. The mean parity of the women was 4.85 (SD = 2.57) having minimum one and maximum thirteen children. More than half of the participants (57.3%) were from terai (plain) region and rest from hilly and mountain regions. More than two third (68.2%) of the patients had travelled more than four hours, with available means of transportation, to reach the diagnostic center. The median travel time to reach the place of diagnosis for cervical cancer was five hours having range from one to 36 hours from patient’s residence.

**Table 1:** Characteristics of the patients of cervical cancer

**Characteristics**	**Number (n)**	**Percentage**
Age at diagnosis (in years)		
Less than 50	41	37.3
50 or more	69	62.7
Mean age ± S.D. = 52.72 ±10.63, Median: 51.50, range 27-79		
Education status		
Illiterate	73	66.4
Literate	37	33.6
Residence		
Urban	25	22.7
Rural	85	77.3
Marital status		
Married and living together	88	80.0
Widow	22	20.0
Parity		
≤3^rd^ parity	35	31.8
> 3^rd^ parity	75	68.2
Mean Parity ± S.D. = 4.85 ± 2.57, range 1-13		
Ecological region of residence		
Terai (plain)	63	57.3
Hills and mountain	47	42.7
Remoteness of place of diagnosis (travel time in Hours)		
<4	35	31.8
≥4	75	68.2
Median travel time = 5 hrs, range 1-36 hrs		

### Diagnostic journey of patients

Table 2 presents the diagnostic journey of cervical cancer patients. The common earlier symptoms were foul smelling vaginal discharge, lower abdominal pain and abnormal PV bleeding including post coital bleeding (PCB), inter- menstrual bleeding (IMB) and post- menopausal bleeding (PMB). Although, more than half (54.5%) of the patients had foul smelling vaginal discharge as the earliest symptom, abnormal vaginal bleeding was the major chief complaint (62.7%) of women that led them to consult a health care provider (HCP) for the first time.

**Table 2:** Description of the history of diagnostic journey of cervical cancer patients

**Characteristics**	**Number (n)**	**Percentage**
Type of earlier symptom		
Foul smelling PV discharge	60	54.5
Lower abdominal pain	25	22.7
Abnormal PV bleeding	25	22.7
Chief complaints to consult HCP		
Foul smelling PV discharge	19	17.3
Lower abdominal pain	22	20.0
Abnormal PV bleeding	69	62.7
Type of first contact health facilities		
SHP/HP/PHC	20	18.2
Private medical shops	37	33.6
Government Hospital	19	17.3
Private hospitals	34	30.9
Number of HFs contacted before being referred to cancer diagnostic center		
Single HF	14	12.7
2-3 HFs	76	69.1
>3 HFs	20	18.2
Mean ± S.D. = 2.7 ± 1.07, range 1-6		
Number of pre-referral consultations in different HFs		
≤ 3	19	17.3
>3	91	82.7
Mean ± S.D. = 4.77 ± 1.8, range 1-10		
Cervical/per speculum examination in initial consultation		
Yes	24	21.8
No	86	78.2
Symptoms misinterpretation in initial consultation with HCP		
No	11	10.0
Yes	99	90.0
Number of visits in cancer diagnostic center till diagnosis		
≤ 2 visits	55	50.0
>2 visits	55	50.0
Mean ± S.D. = 2.65 ± 0.77, range 2-5		
Stage of cervical cancer at diagnosis (FIGO)		
IB1	10	9.1
IB2	5	4.5
IIA	6	5.5
IIB	56	50.9
IIIA	3	2.7
IIIB	29	26.4
IVA	1	0.9

Private medical shops (33.6%) and private hospitals (30.9%) were the main primary contact point with a health care provider for the patients. Government’s community level health facilities: Sub-Health Post/Health Post/Primary Health Center (SHP/HP/PHC) and hospitals were reported as the primary contact point only in 18.2% and 17.3% respectively. In average women had consulted about three different health facilities and most of them (82.7%) had more than three pre-referral consultations with HCP.

Cervical examination was not performed in more than three fourth (78.2%) and most of their (90.0%) symptoms were misinterpreted in initial consultation with HCP. The average number of pre-diagnostic visits of the patients in cervical cancer diagnostic center was 2.65 (SD = 0.77) with range two to five. The clinical staging of the cervical cancer was done according FIGO staging system update 2009 [21]. Only 19% of the patients were diagnosed in early stage (≤IIA) and rest were in advantaged stage (≥IIB).

### Estimates of delays in diagnosis

The median patient delay was 68 days with long patient delay in 57% of the patients. Similarly, median HCP delay was 40 days with 80.9% patients having long HCP delay. The referral delay was found comparatively low (median 5 days) with more than two third (68.2%) having short referral delay. After the first visit to diagnostic center, two third (66.2%) of the patients had to wait more than one week for diagnosis of cervical cancer (median diagnostic waiting time of nine days). The median value of total diagnostic delay was 157 days with longer total diagnostic delay in 77.3% of patients (Table 3).

**Table 3:** Extent of patient delay, health care provider delay, referral delay, diagnosis waiting time and total diagnostic delay in cervical cancer diagnosis by selected patient characteristics and other attributes

**Attributes**	**Patient delay**			**Health care provider delay**			**Referral delay**			**Diagnostic waiting time**			**Total diagnostic delay**		
	**Median (min- max)**	**Short delay n (%)**	**Longer delay n (%)**	**Median (min-max)**	**Short delay n (%)**	**Longer delay n (%)**	**Median (min-max)**	**Short delay n (%)**	**Longer delay n (%)**	**Median (min- max)**	**Short delay n (%)**	**Longer delay n (%)**	**Median (min-max)**	**Short delay n (%)**	**Longer delay, n (%)**
Age in years															
Less than 50	66 (8-306)	20 (48.8)	21 (51.2)	41 (0-372)	1 (2.4)	40 (97.6)	5 (0-49)	27 (65.9)	14 (34.1)	8 (3-40)	18 (43.9)	23 (56.1)	148 (36-622)	12 (29.3)	29 (70.7)
Fifty or more	71 (8-404)	27 (39.1)	42 (60.1)	39 (0-582)	10 (14.5)	59 (85.5)	5 (0-88)	48 (69.6)	21 (34.4)	9 (2-57)	19 (27.5)	50 (72.5)	160 (22-718)	13 (18.8)	56 (81.2)
Education															
Illiterate	72 (8-353)	27 (37.0)	46 (63.0)	41 (0-582)	6 (8.2)	67 (91.8)	5 (0-88)	46 (63.0)	27 (37.0)	9 (2-57)	23 (31.5)	50 (68.5)	158.7 (25-718)	13 (17.8)	60 (82.2)
Literate	48 (9-404)	20 (54.1)	17 (49.9)	21 (0-282)	5 (13.5)	32 (86.5)	3 (0-32)	29 (78.4)	8 (21.6)	8 (3-40)	14 (37.8)	23 (62.2)	143 (22-431)	12 (32.4)	25 (67.6)
Remoteness (travel time)															
Less than 4 hours	48 (8-274)	19 (54.3)	16 (45.7)	16 (0-282)	5 (14.3)	30 (85.7)	2 (0-18)	30 (85.7)	5 (14.3)	8 (2-57)	15 (42.9)	20 (57.1)	123 (25-376)	12 (34.3)	23 (65.7)
Four hours and more	72 (8-404)	28 (37.3)	47 (62.7)	49 (0-582)	6 (8.0)	69 (92.0)	6 (0-88)	45 (60.0)	30 (40.0)	9 (3-38)	22 (29.3)	53 (70.7)	177 (22-718)	13 (17.3)	62 (82.7)
Types of earlier symptoms															
Vaginal discharge	71.5 (8-404)	23 (38.3)	37 (61.7)										199 (25-622)	7 (11.7)	53 (88.3)
Pelvic pain	62 (9-239)	12 (48.0)	13 (52.0)										146 (36-718)	9 (36.0)	16 (64.0)
Abnormal vaginal bleeding	65 (10-304)	12 (48.0)	13 (52.0)										106 (22-403)	9 (36.0)	16 (64.0)
Chief complaints															
Vaginal discharge				69 (9-582)	0 (0.0)	19 (100.0)							215 (69-718)	2 (10.5)	17 (89.5)
Pelvic pain				38.5 (6-316)	1 (4.5)	21 (95.5)							144 (36-631)	5 (22.7)	17 (77.3)
Abnormal vaginal bleeding				25 (0-372)	10 (14.5)	59 (85.5)							153 (22-622)	18 (26.1)	51 (73.9)
Type of first contact health facilities															
SHP/HP/PHC				72.5 (10-582)	0 (0.0)	20 (100.0)							214 (56-718)	4 (20.0)	16 (80.0)
Private medical shops				64 (7-316)	3 (8.1)	34 (91.9)							158 (48-631)	8 (21.6)	29 (78.4)
Government hospitals				38 (0-348)	3 (15.8)	16 (84.2)							142 (36-430)	5 (26.3)	14 (73.7)
Private hospitals				19 (0-217)	5 (14.7)	29 (85.3)							137 (22-374)	8 (23.5)	26 (76.5)
Pre-referral consultations with HCP															
Upto three				11 (0-133)	8 (42.1)	11 (57.9)							112 (22-428)	6 (31.6)	13 (68.4)
More than three				49 (6-582)	3 (3.3)	88 (96.7)							160 (25-718)	19 (20.9)	72 (79.1)
Per-Speculum examination in initial consultation															
Yes				9.5 (0-151)	8 (33.3)	16 (66.7)							108 (22-428)	9 (37.5)	15 (62.5)
No				53.5 (6-582)	3 (3.5)	83 (96.5)							168 (36-718)	16 (18.6)	70 (81.4)
Total	68.5 (8-404)	47 (42.73)	63 (57.27)	40 (0-582)	11 (10.0)	99 (90.0)	5 (0-88)	75 (68.2)	35 (31.8)	9 (2-57)	37 (33.6)	73 (66.4)	157 (22-718)	25 (22.7)	85 (77.3)

Table 3 presents the details of patient delay, health care provider delay, referral delay, diagnostic waiting time and total diagnostic delay distribution according to selected patient’s characteristics and other attributes. The long total diagnostic delay (>90 days) was observed more among patients aged 50 years or more (81.2%), illiterate (82.2%) and those residing at remote places (82.7%). Long patient delay (median 71.5 days) and total diagnostic delay (median 199 days) were found in patients with early symptoms like foul smelling vaginal discharge as compared to patients with other symptoms. The longer total diagnostic delay was also higher (88.3%) among these women. Cent percent longer health care provider’s delay with median 69 days and higher proportion of total diagnostic delay (89.5%) with median delay of 215 days was observed among patients who consulted HCP for the first time with chief complaint of foul smelling vaginal discharge. These statistics were comparatively lower among patients with chief complaints of pelvic pain and abnormal vaginal bleeding. Similarly, longer HCP delay (median 72.5 days) and total diagnostic delay (median 214 days) were observed among women whose primary contact point was community level health (SHP/HP/PHC). Higher proportion of HCP delay (96.7%) and total diagnostic delay (79.1%) were measured among patients who had more than three pre-referral consultations. The HCP delay and total diagnostic delay were found higher among the patients whose cervix was not examined in initial consultation.

## Discussion

This study identified different lag periods in diagnosis of cervical cancer, total diagnostic delay categorized into patient delay, healthcare provider delay, referral delay and diagnostic waiting time. Although, no standardized definition of delay is found, the studies on diagnostic delays have several common themes regarding the length of delay based on dates of important events in diagnostic journey of the patients [7,13,14,20]. Similar type of component delays of cancer diagnosis have been applied in previous studies [13,14,22]. Variation can be found in the point of dichotomization of each type of delay into long and short delay but it is very contextual [9,13,23-25]. Hansen has categorized delays as short or long based on quartiles and described delays in terms of median and inter-quartile range [13]. Other studies have considered certain time periods such as days, weeks or months for this purpose [20,23-26].

This study revealed the median patient delay of 68.5 days, median health care provider delay of 40 days, median referral delay of 5 days, median diagnostic time of 9 days and median total diagnostic delay of 157 days. This diagnostic delay was found higher in Nepal when compared with that of developed countries [24,26]. High prevalence of long diagnostic delay of more than three months maybe extremely unacceptable if cancer is to be treated in early stage. The longer duration of symptoms till diagnosis supports the high prevalence of late stage diagnosis of cervical cancer in Nepal [11,12]. Patient and health care provider delay accounted for most of the total delay. As a major delay, Nepalese women had suffered longer patient delay with the wide range of 8 – 404 days, as compared to other delays. The longer patient delay in Nepal may have resulted due to the influence of patient’s characteristics such as high level of illiteracy, poor health awareness, poor economic condition, their problematic health seeking behavior, ignoring the mild gynecological symptoms as well as dependency on traditional health care practices [9,12]. Previous studies have also revealed that the patient’s behavior of not recognizing symptom seriousness and rather ignoring them as the factors for increasing patient delay in diagnosis of cervical cancer [9,23]. In some population, prevalent symptom like vaginal discharge is not recognized as warning symptom and in most cases medical assistance is not sought until it becomes obvious and unbearable, eventually leading to longer patient delay and total diagnostic delay [27,28].

This study establishes the fact that health care provider’s delay as another major delay in cervical cancer diagnosis. Although in low proportion, longer medical delay have also been observed in previous studies, even in developed countries [13,23]. The observation of medical delay in Morrocco where 61% of patients had suffered ≥30 days’ delay was similar to that of Nepal. The high proportion of health care provider delay in Nepal can be argued from various perspectives such as access to services, education level of health care providers and existing health care system and policy. In Nepal, the first contact point like the sub-health post (SHP) and health post (HP) from public service and private medical shops at community level are run by health care providers having basic medical trainings. These health workers often lack competency on gynecological examination and knowledge on cervical cancer screening and detection. In the existing health care system of Nepal, all woman do not have access to gynecologists or medical doctors for their gynecological symptoms [29]. This argument is in line with the findings that women had to visit many health facilities for several times before being finally referred to diagnostic center. The structure of health care system, referral mechanism, socio-cultural factors, knowledge level of health care provider and asymmetric relationship between health care provider and patients influence the health seeking practices of patients [30,31]. Inadequate knowledge of cervical cancer etiology, alarming symptoms, screening, diagnostic procedure and treatment among health care practitioners contribute in delays in diagnosis [16], eventually leading to the misdiagnosis of cancer. Non-recognition of cervical cancer symptoms and/or not being able to provide a cervical examination by health care provider in initial consultation creates the situation of unnecessary visits in different health institutions [17,20]. Patients who complained of alarming symptoms such as abnormal vaginal bleeding or severe pelvic pain had faced shorter HCP delay as compared to patients with other symptoms like foul smelling vaginal discharge. Gynecological examination by the HCP has been observed to be performed less often in woman without vaginal bleeding. The length of the delay has been reported shortened in patients who had gynecological examination by the HCP for complaints of alarming symptoms [20].

The length and frequency distribution of each type of diagnostic delays varied in different groups of participants. Elderly and illiterate women residing in remote areas had longer patient delay, HCP delay, diagnostic waiting time and total diagnostic delay in high proportion. Studies have also revealed advanced age as a risk factor of patient delay in cancer diagnosis including cervical cancer [9,19,23]. Higher proportion of longer delays in certain groups of patients depicts the barriers in health care access and prolongation in delays in diagnosis among those population [23,28].

## Conclusions

Delay in diagnosis is a major issue in cancer prevention, treatment and control. Longer delays observed all over the diagnostic pathway is of serious concern as this result in high prevalence of advanced stage at diagnosis and high mortality. Among the delays, patient delay is of crucial importance because of its longer span; however health care provider delay is equally important. Thus, education of both the patient and health care providers is essential for early diagnosis. There is a need of comprehensive approach to address two major delays: patient delay and health care provider (HCP) delay by increasing the patient’s awareness, enhancing the health care provider’s capacity for early recognition of cervical cancer symptoms and establishing timely referral mechanism for diagnosis.

## Abbreviations

BPKMCH: BP Koirala Memorial Cancer Hospital; HCP: Health care provider; HP: Health post; IMB: Inter-menstrual bleeding; PCB: Post coital bleeding; PHC: Primary health center; PMB: Post menopausal bleeding; PV: Per Vaginum; SD: Standard deviation; SHP: Sub-health post.

## Competing interests

The authors declare that they have no competing interests.

## Authors’ contributions

DG, AA, SRO: Design of the study; DG, GK: Data collection, data management; DG, GK, RP, JP: Data analysis, writing paper. All authors read and approved the final manuscript.

## Authors’ information

DG is a public health graduate and carried out this study as his thesis project for MPH degree at department of community medicine and public health, Institute of Medicine, Tribhuvan University. GK is a graduate nurse with major in community nursing and she is working as a nursing officer at Bharatpur hospital, Chitwan. RP is lecturer of public health at Institute of Medicine, Tribhuvan University. AA is an Associate Professor of public health at Institute of Medicine. JP is a gyaenecologic oncologist at BP Koirala memorial cancer hospital, Chitwan. SRO is an assistant Dean of Institute of Medicine and Professor of public health at department of community medicine and public health, Institute of Medicine, Tribhuvan University.

## Pre-publication history

The pre-publication history for this paper can be accessed here:

http://www.biomedcentral.com/1472-6874/14/29/prepub
